# Silybin Derivatives Produced by γ-Irradiation and Their Tyrosinase Inhibitory Activities

**DOI:** 10.3390/molecules29225332

**Published:** 2024-11-13

**Authors:** Ah-Reum Han, Hyung Won Ryu, Chang Hyun Jin

**Affiliations:** 1 Advanced Radiation Technology Institute, Korea Atomic Energy Research Institute (KAERI), Jeongeup-si 56212, Jeonbuk-do, Republic of Korea; 2 Natural Medicine Research Center, Korea Research Institute of Bioscience & Biotechnology (KRIBB), Cheongju-si 28116, Chungbuk-do, Republic of Korea

**Keywords:** silybin, isosilandrin, 2,3-dehydrosilybin, radiation, tyrosinase, molecular docking

## Abstract

Silybin, which belongs to the flavonolignan group, is the major component of the fruit extract of *Silybum marianum* (common name: milk thistle). Silybin is a medicinal compound with hepatoprotective, antioxidant, and anticancer properties. In this study, silybin derivatives were produced through γ-radiolysis, and their tyrosinase inhibitory activities were evaluated to explore the enhanced activities of silybin derivatives compared to silybin (**1**). Isosilandrin (**2**) and 2,3-dehydrosilybin (**3**) were obtained from a silybin sample irradiated at 300 kGy. The optimal dose showed significant changes in radiolysis product content. Compounds **2** and **3** exhibited an IC_50_ of 274.6 and 109.5 μM, respectively, which are more potent than that of **1** (IC_50_ > 500 μM). In addition, a molecular docking simulation revealed the binding affinity of these compounds to tyrosinase and their mechanisms of inhibition. Thus, γ-irradiation is an effective method for structural modification of silybin. We also demonstrated that 2,3-dehydrosilybin is a potential tyrosinase inhibitor.

## 1. Introduction

*Silybum marianum*, also known as milk thistle, is a member of the Asteraceae family and this extract has been used for centuries as a traditional folk medicine for liver protection in Europe and United States of America [1]. Silymarin, a mixture of flavonolignans extracted from the seeds of *S. marianum*, has demonstrated a broad spectrum of biological activities, including hepatoprotective [2], antidiabetic [3], anti-inflammatory [4], and antiviral effects [5]. The major component of silymarin is known to be silybin (synonym: silibinin; ca. 30%), which occurs naturally as a mixture of two diastereoisomers, silybin A and silybin B in approximately 1:1 ratio [6]. Other flavonolignans present in lesser amounts are isosilybin, sylimonin, and 2,3-dehydrosilybin [7]. These compounds have been isolated from the violet-flowered *S. marianum*, while the other flavonolignans, silandrin and isosilandrin, were found in the white-flowered *S. marianum* [8,9,10].

To introduce a new synthesis method for silybin derivatives, we irradiated silybin with gamma rays and confirmed the irradiation-induced formation of its derivatives. The modification of molecular structures using gamma-radiation is an economical, eco-friendly, and convenient method for obtaining derivatives with new or improved functionality [11]. Recently, there have been reports on the use of ionizing radiation for the production of new derivatives and the development of natural product compounds with improved bioactivity [11], such as genistein [12], chrysin [13], and 4-methylumbelliferone [14]. Related to the synthesis of individual flavonolignans in *S. marianum*, silybin was originally synthesized starting from taxifolin and coniferyl alcohol by horseradish-peroxidase and feeding suspension culture [15]. The total synthesis of isosilandrin has been previously reported [10], and 2,3-dehydrosilybin has been simply produced from silybin by an oxidation method [16,17,18,19]. However, the structural modification of silybin using ionizing irradiation has not been studied.

Melanin synthesis is usually initiated by ultraviolet (UV) light. However, excessive pigmentation caused by melanin overproduction leads to numerous skin problems including age spots and malignant melanoma [20]. The key enzyme involved in melanogenesis is tyrosinase [21]. This enzyme catalyzes two different processes involving the oxidation of tyrosine to 3,4-dihydroxyphenylalanine and then to dopaquinone [22]. Therefore, research on the inhibition of tyrosinase for antipigmentation has been attracting increasing attention in pharmaceutical and cosmetic industries, and discoveries of new tyrosinase inhibitors have been focused on natural products [23]. Interestingly, there have been reports on silybin-inhibited monophenolase and diphenolase of tyrosinase [24], and silybin showed inhibitory effects on melanogenesis and proliferation in B16 melanoma 4A5 cells [25]. In addition, silybin induced melanogenesis through the protein kinase A and p38 mitogen-activated protein kinase (MAPK) signaling pathways in B16-F1 melanoma cells [26].

This study aimed to apply ionizing radiation to silybin for molecular modification and to examine the bioactivity enhancement of the radiolytic products of silybin. Silybin derivatives were isolated and their tyrosinase inhibitory activities were evaluated. In addition, molecular docking simulation was employed to predict the binding affinity of the diastereomers of the given compounds in the active site of mushroom tyrosinase.

## 2. Results and Discussion

### 2.1. Structure Identification of Two Radiolysis Products Derived from Silybin

**Figure 1:**
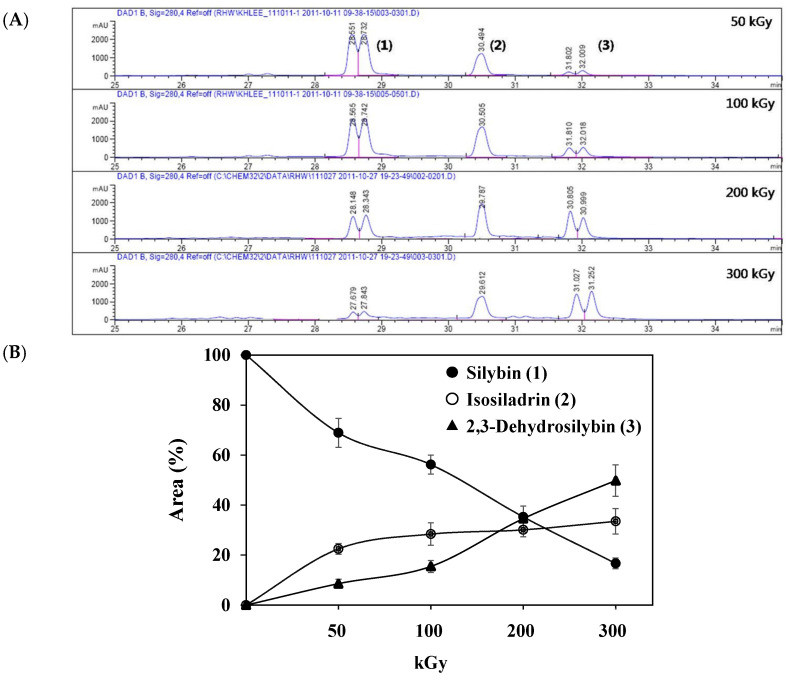
(**A**) HPLC profiles of silybin (**1**; the mixture of silybin A and silybin B) irradiated with the following doses: 50, 100, 200, and 300 kGy. The HPLC chromatogram was detected at 280 nm (UV). Peak 1: silybin (**1**); Peaks 2 and 3: radiolysis products of **1**. (**B**) Kinetic analysis of the conversion of peak 1 to peaks 2 and 3.

**Figure 2:**
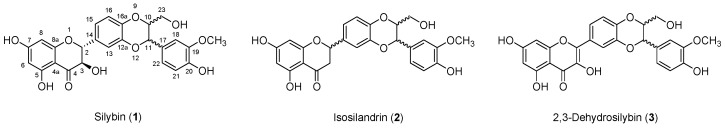
The chemical structures of compounds **1**–**3**.

The sample solutions containing silybin (**1**; the mixture of silybin A and silybin B) were irradiated at different doses: 50, 100, 200, and 300 kGy. Their degradation patterns were then confirmed through HPLC-DAD analysis. Under this LC analysis condition, the peaks of silybin, which is a 1:1 mixture of silybin A and silybin B, appeared as two peaks, and peak 3, the radiolysis product, also appeared as two peaks as it converted into a mixture, whereas peak 2, though a mixture, did not split into two peaks but appeared as one broad peak. As the radiation dose increased, the peak area of silybin decreased while the peak area for the radiolysis products increased (Table 1 and Figure 1). At a radiation dose of 300 kGy, the two radiolysis products from silybin were produced with the highest yield, and their relative contents were higher than 16.7% of peak area of silybin, increasing to 33.5 and 49.8% of the peak area, respectively. The application of medium pressure liquid chromatography (MPLC) to this sample led to the isolation of two compounds **2** and **3**. Their structures were identified by several NMR techniques, including ^1^H, ^13^C, DEPT, ^1^H-^1^H COSY, ^1^H-^13^C HSQC, ^1^H-^13^C HMBC nuclear magnetic resonance (NMR), and high-resolution electron ionization mass spectroscopy (HREIMS) data. Compound **2** was obtained as a colorless powder and exhibited a molecular ion at m/z 466.1265 [M]^+^ in HREIMS, corresponding to a molecular formula of C_25_H_22_O_9_. ^1^H and ^13^C spectra of **2** were similar to those of silybin (**1**), except for the presence of characteristic signals for flavanone at δ_H_ 2.76 (1H, m, H_eq_-3), 3.13 (1H, dd, *J* = 17.2, 12.7 Hz, H_ax_-3), and δ_C_ 42.6 (C-3), instead of signals for a hydroxymethyl group positioned at C-3 in 1. Thus, **2** was identified as isosilandrin, by comparing its spectroscopic data with literature values [10] (Figure 2). Compound **3** was obtained as a yellowish powder and gave a molecular ion peak at m/z 386.1368 [M]^+^ in HREIMS, corresponding to an elemental formula of C_25_H_20_O_10_. A comparison of the ^1^H and ^13^C spectral data of **3** with those of **1** indicated the absence of signals for two hydroxy methane groups and the presence of two quaternary carbon signals at δ_C_ 136.7 (C-3) and 146.1 (C-2) in **3**. Based on a further detailed analysis of 2D NMR data and by comparison of its spectral data with literature values [18], **3** was identified as 2,3-dehydrosilybin (Figure 2).

The total synthesis of isosilandrin (**2**) has been reported with a yield of 2.64% (dry weight) of the starting material, ethyl ester (2*E*)-3[(2*R*,3*R*)-2,3-dihydro-3-(4-hydroxy-3-methoxyphenyl)-2-(hydroxymethyl)-1,4-benzodioxin-6-yl-2-propenoic acid, by condensation of a derivative from the starting compound and 2-hydroxy-4,6-bis(methoxy)acetophenone [10]. Meanwhile, 2,3-dehydrosilybin has been simply produced from silybin by the oxidation method, including treatment with hydrogen peroxide in a solution of sodium bicarbonate [17], reaction with pyridine at reflux [18], and reaction with potassium acetate in dimethylformamide [19]. In this study, compounds **2** and **3** were purified to 335 mg and 412 mg, respectively, from a sample irradiated with the optimal dose of 300 kGy for 1 g of silybin, with yields of 33.5% and 41.2%, respectively. Therefore, the semi-synthesis of compound **2** using ionizing radiation proves much more efficient than its total synthesis (yield of 2.64%). Compound **3** was produced with a yield of 51% by the oxidation method [18]; meanwhile, a yield of 41.2% was obtained by using ionizing radiation, which is as simple as the oxidation method. Although silybin and other flavonolignans have been synthesized using chemical- or synthase-based methods, there has been no report on their structural modification by ionizing radiation.

### 2.2. Inhibition Effects of Mushroom Tyrosinase

Five sample solutions containing silybin irradiated with 0, 50, 100, 200, and 300 kGy were tested for enzymatic inhibitory activities against tyrosinase, comparing them to the positive control arbutin, which is a well-known tyrosinase inhibitor. The irradiated silybin samples exhibited tyrosinase inhibitory activity in a dose-dependent manner, and their IC_50_ values were significantly better than that of arbutin (Table 2). Among them, the 300 kGy irradiated sample with the highest radiolysis product content showed the highest potency on tyrosinase inhibition with an IC_50_ value of 171.1 μg/mL and showed a 13-fold stronger inhibitory activity than arbutin (IC_50_ = 2300 μg/mL) [27]. A further analysis of the tyrosinase activity showed that isosilandrin (**2**) and 2,3-dehydrosilybin (**3**) demonstrated significant inhibitory activity with IC_50_ values of 274.6 and 109.5 μM, respectively, compared to silybin (**1**) (Table 3). The difference in the structures of these three compounds is based on the oxidation or reduction of the C-ring in the phenylchromane skeleton of the flavonoid. It was assumed that 3 showed the greatest inhibitory activity due to the presence of an unsaturated conjugation system in the C-ring, which was structurally transformed to interact with the enzyme [28,29].

Silybin (**1**) has been reported to inhibit tyrosinase by mixed type I (K_I_ < K_IS_) inhibition against both monophenolase and diphenolase [24]. Moreover, studies on silybin’s melanin induction and mechanism of action revealed that silybin stimulates melanogenesis through the activation of protein kinase A and p38 MAPK-dependent pathways, leading to cyclic AMP-responsive element-binding protein phosphorylation and microphthalmia-associated transcription factor expression [26]. Therefore, the enhanced tyrosinase inhibitory activities of radiolysis products **2** and **3** suggest that these compounds could be more therapeutically useful than silybin.

Among the few biological activities of isosilandrin (**2**) is the inhibition of the superoxide anion release by human polymorphonuclear leukocytes [10] and the production of nitric oxide through the suppression of nuclear factor kappa B (NF-κB) transcriptional activation [30]. Meanwhile, 2,3-dehydrosilybin (**3**) has diverse biological activities. For instance, it exhibits greater antioxidant activity compared with silybin by protecting H_2_O_2_-induced HepG2 cell death and anticancer activity by inhibiting metalloproteinases-2 and -9, which are responsible for invasive metastasis [31]. Compound **3** also has multidrug resistance modulation activities: it inhibits the P-glycoprotein pump for the sensitization of doxorubicin-resistant ovarian carcinoma [32]; and it modulates the antibiotic resistance of Staphylococcus aureus and reduces its virulence [33]. In addition, compound **3** also modulates skin damage response. For instance, compound **3** modulated the pro-inflammatory cytokines production via the NF-κB and activator protein 1 signaling pathways [34]. Compound **3** also reduced inflammation and supported epidermis regeneration through downregulating the production of selected pro-inflammatory cytokines produced by normal human epidermal keratinocytes [35]. Moreover, it has suppressed UVA-caused oxidative stress of human keratinocytes HaCaT [36]. However, compounds **2** and **3** have been not reported to have any inhibitory activity with respect to tyrosinase, an enzyme important for melanin synthesis through melanogenesis.

### 2.3. Molecular Docking Analysis of Silybin Derivatives

**Figure 3:**
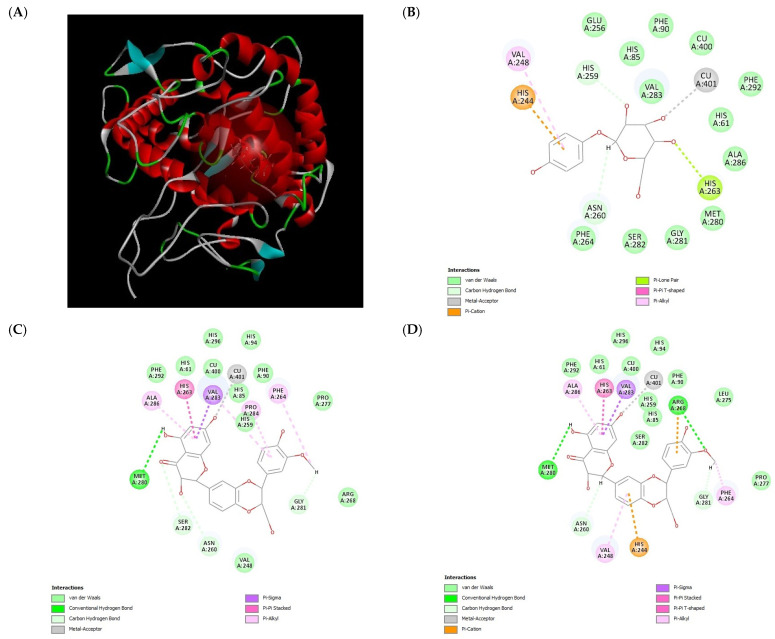

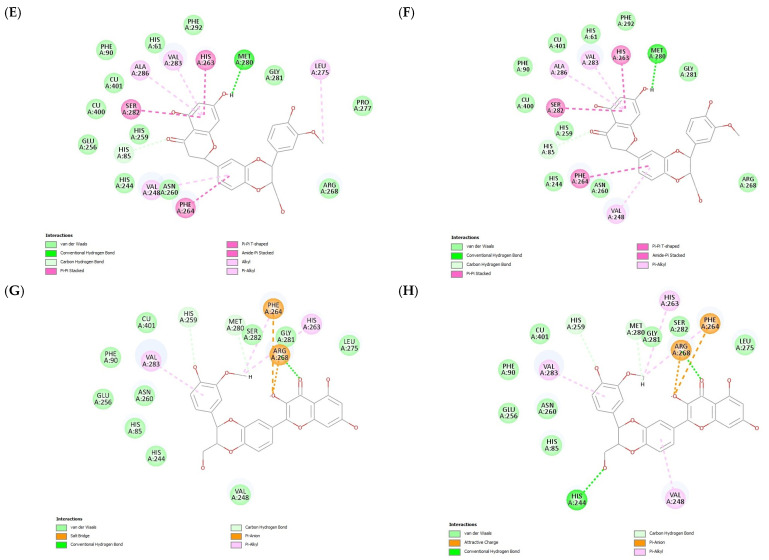
(**A**) A chain of mushroom tyrosinase and favorable pose of arbutin (the positive control) in binding site. Two-dimensional receptor–ligand interacting modes of (**B**) arbutin, (**C**) (+)-silybin A, (**D**) (−)-silybin B, (**E**) (−)-isosilandrin A, (**F**) (+)-isosilandrin B, (**G**) (−)-dehydrosilybin, and (**H**) (+)-dehydrosilybin B in the active site of mushroom tyrosinase.

Based on the suggested tyrosinase inhibition and melanin induction mechanism of silybin, we examined possible binding interactions between the selected structures of silybin derivatives and mushroom tyrosinase. For docking, we used the X-ray crystal structure (PDB ID: 2Y9X) of mushroom tyrosinase (*Agaricus bisporus*) complexed with tropolone. The structure was obtained from the RCSB Protein Data Bank (PDB) [37]. The A chain of this protein has five binding active sites (AC1, AC2, AC3, AC4, and AC7). AC1 consists of His61, Cys83, His85, and His94; AC2 consists of His259, His263, and His296; AC3 consists of Asn260, His263, Phe264, Met280, Val283, Ala286, and Cu401; AC4 consists of Asp336, Gln351, and Asp353; and AC7 consists of Asp312. The molecular docking experiments of the selected stereostructures of **1**–**3**, (+)-silybin A, (−)-silybin B, (−)-isosilandrin A, (+)-isosilandrin B, (−)-dehydrosilybin, and (+)-dehydrosilybin B, within the active binding site of tyrosinase were conducted using the Discovery Studio software ver. 21.0.1.20298 (BIOVIA/Accelrys Inc., San Diego, CA, USA). The docking score and interaction results are listed in Table 4. Six compounds showed stable binding energy levels with tyrosinase with –CDOCKER energies larger than that of arbutin (5.0142 kcal/mol). Arbutin was located partially in AC2 and AC3, with the interactions of His259, His263, Asn260, Cu401, His244, and Val248 (Figure 3). This finding was similar to the molecular docking result of arbutin in a previous report [38,39]. The values of –CDOCKER energy of (+)-silybin A and (−)-silybin B were greater than that of arbutin and similar to each other (Table 4). The chromanone core in their structures interacted closely with the key residues of site AC3, namely Met280, Ala286, His263, Val283, Asn260, and Cu401 (Figure 3). Phe264 and Gly281 formed an interaction with the methoxy group substituted on the phenylpropane moiety. Moreover, (−)-Isosilandrin A and (+)-isosilandrin B had slightly higher –CDOCKER energy values to (+)-silybin A and (−)-silybin B. While these two also showed similarity in that they were partially located in AC3 and interacting with the residues Met280, Ala286, His263, and Val283, there were differences showing the interaction of His85 and the carbonyl group in flavanone at site AC1, Phe 264, Val 248, and the C-ring in flavanone (Table 1 and Figure 3). Furthermore, (−)-dehydrosilybin, and (+)-dehydrosilybin B, which have flavonol structures, docked into the AC2 and AC3 pockets with the highest values of –CDOCKER energy compared to the five previously docked compounds. The difference in the docking poses explained the difference in interactions compared with the findings for silybin and isosilandrin. The phenylpropane moiety interacted with the residues of sites AC1 and AC3, namely His259, Met280, His263, and Val283. The 3-hydroxy group located on flavonol formed interactions with Arg268 and Phe264. In a comparison of the docking simulation data with the results of the experimental in vitro assay for mushroom tyrosinase inhibition, a good correlation was found between the docking scores and IC_50_ values of docked compounds. The explanation for this could be that the structurally different poses of (−)-dehydrosilybin and (+)-dehydrosilybin B in the active sites resulted in the higher inhibitory activity of those compounds against tyrosinase. In addition, the inhibition constant (K*i*) was calculated from the binding energy (ΔG) using the following formula: K*i* = exp(ΔG/RT) [R: universal gas constant (1.985 × 10^−3^ kcal mol^−1^ K^−1^); T: temperature (298.15 K)] [40]. In the case of arbutin, the K*i* value was 209.2 μM, but the K*i* values of the remaining compounds were too low (fM or less) to be presented in Table 4. In a report on the theoretical analysis of the relationship between the K*i* of a compound and the IC_50_ value, which represents the inhibitor concentration required to inhibit the enzyme reaction by 50% at a specific substrate concentration [41], the authors explain that K*i* is not equal to IC_50_ when competitive inhibition kinetics are applied, but K*i* is equal to IC_50_ under noncompetitive or uncompetitive kinetic conditions. It suggests that the silybin derivatives that were treated as structurally similar compounds to the substrate showed a behavior with competitive inhibition, competing for binding to the active site of the enzyme or preventing the substrate from binding to the active center of the enzyme.

## 3. Materials and Methods

### 3.1. Methods

NMR spectra were performed using a Bruker AM500 instrument (^1^H NMR at 500 MHz, ^13^C NMR at 125 MHz, Billerica, MA, USA) and JEOL ECX-500 instrument (^1^H NMR at 500 MHz, ^13^C NMR at 125 MHz, JEOL, Tokyo, Japan). EI-HR-MS (JEOL JMS-700, JEOL Ltd., Tokyo, Japan) was used to collect spectroscopic data. An analytical HPLC-DAD-MSD process was carried out on an Agilent 1100 series system (Agilent Technologies Co., Santa Clara, CA, USA) and an Agilent mass spectrometer detector equipped with an electrospray ionization (ESI) source (Agilent Technologies Co., Santa Clara, CA, USA), equipped with a TSK-GEL ODF-100 V column (4.6 × 150 mm, 5 μm; TOSOH, Tokyo, Japan). Separations were conducted using RediSep Rf Normal Phase Silica columns on MPLC instrument (Teledyne ISCO CombiFlash^®^, Lincoln, NE, USA). Thin layer chromatography (TLC) was performed on precoated TLC plates using Kieselgel 60 F254 (Merck, Darmstadt, Germany). These were visualized at 254 nm using a UV lamp (UVP, Cambridge, UK), and 10% sulfuric acid spray was applied followed by heating. DMSO-*d*_6_ and acetone-*d*_6_ were purchased from Cambridge Isotope Lab. Inc., (Tewksbury, MA, USA).

### 3.2. Sample Preparation

Irradiation was carried out at ambient temperature, using a [^60^Co] γ-irradiator (150 TBq capacity; AECL, Ottawa, ON, Canada) in the Advanced Radiation Technology Institute, Korea Atomic Energy Research Institute (Jeongup-si, Korea). The source intensity was approximately 320 kCi, and the dose rate at the location of the sample was 10 kGy/h. Dosimetry was performed using alanine dosimeters (Bruker Instruments, Rheinstetten, Germany) with a diameter of 5 mm, which were calibrated according to the International Standard set by the International Atomic Energy Agency (Vienna, Austria). Silybin (the racemic mixture) was purchased from Sigma-Aldrich Co. (St. Louis, MO, USA). Sample solutions (1 g silybin in 100 mL methanol in 10% DMSO) was divided into 100 vials, which were then irradiated with 300 kGy (absorbed dose). After γ-irradiation, the sample solution was immediately evaporated to remove the solvent and freeze-dried for further analysis.

### 3.3. HPLC DAD-MSD

An analysis of the γ-irradiated sample solutions (1 g silybin in 100 mL methanol in 10% DMSO) was performed by HPLC-DAD-MSD analysis with a TSK-GEL ODF-100 V column (4.6 × 150 mm, 5 μm; TOSOH). Gradient elution was carried out with 0.5% formic acid in water (A) and acetonitrile (B). The gradient elution program was as follows: 0.5 min, 0% B; 15 min, 65% B; 35 min, 75% B; 65 min, 85% B; and 85 min, 100% B, and then held for 15 min before returning to the initial conditions. Samples (5 μL) were injected at a flow rate of 1 mL/min. Chromatograms were acquired at 280 nm. ESI was performed in positive ion mode with the following parameters: capillary potential, 4000 V; drying gas temperature, 350 °C; gas flow (N_2_), 12 L/min; and nebulizer pressure, 35 psig. Data were acquired for a mass scan range of *m*/*z* 100 to 1000.

### 3.4. Isolation of Silybin Derivatives

The γ-irradiated silybin (1 g) was subjected to MPLC with a reversed-phase silica gel cartridge column. The mobile phase was composed of water (A) and methanol (B). The gradient conditions were as follows: 30 min, 40% B; 70 min, 50% B; 90 min, 100% B. The total flow rate was maintained at 20 mL/min, and chromatograms were acquired at 280 nm. Radiolysis products **2** (335 mg, 33.5% yield) and **3** (412 mg, 41.2% yield) were purified.

Isosilandrin (**2**). Colorless powder. ^1^H NMR (500 MHz, acetone-*d*_6_) δ 2.76 (1H, m, H_eq_-3), 3.13 (1H, dd, *J* = 17.2, 12.7 Hz, H_ax_-3), 3.54 (1H, m, H-23), 3.72 (1H, m, H-23), 3.84 (3H, s, OCH_3_), 4.11 (1H, dd, *J* = 7.9, 3.7 Hz, H-10), 4.96 (1H, d, *J* = 7.9 Hz, H-11), 5.41 (1H, dd, *J* = 12.7, 3.2 Hz, H-2), 5.93 (1H, d, *J* = 2.0 Hz, H-6), 5.95 (1H, d, *J* = 2.0 Hz, H-8), 6.86–7.10 (6H, m, H-21, 22, 18, 16, 13, 15), 7.94 (s, 20-OH), 12.13 (s, 5-OH); ^13^C NMR (125 MHz, acetone-*d*_6_) δ 42.6 (C-3), 55.6 (18-OCH_3_), 61.1 (C-11), 76.4 (C-13), 78.3 (C-12), 78.8 (C-2), 95.1 (C-6), 96.1 (C-8), 102.5 (C-10), 111.1 (C-15), 115.0 (C-19), 116.9 (C-6′), 116.9 (C-5′), 119.7 (C-16), 120.8 (C-1′, 2′), 128.3 (C-14), 132.8 (C-2), 144.1 (C-3′, 4′), 147.2 (C-17), 147.7 (C-18), 163.4 (C-9), 164.4 (C-5), 166.5 (C-7), 196.2 (C-4); EIMS *m*/*z* 466 [M]^+^; HREIMS *m*/*z* 466.1265, (calcd for C_25_H_22_O_9_, 466.1264).
2,3-Dehydrosilybin (**3**). Yellowish powder. ^1^H NMR (500 MHz, DMSO-*d*_6_) δ 3.38 (1H, br d, *J* = 12.3 Hz, H-23), 3.58 (1H, br d, *J* = 12.3 Hz, H-23), 3.80 (3H, s, OCH_3_), 4.28 (1H, ddd, *J* = 7.8, 4.7, 1.5 Hz, H-10), 4.97 (1H, d, *J* = 7.8 Hz, H-11), 6.20 (1H, d, *J* = 1.9 Hz, H-6), 6.46 (1H, d, *J* = 1.9 Hz, H-8), 6.83 (1H, d, *J* = 8.0 Hz, H-21), 6.90 (1H, dd, *J* = 8.0, 1.1 Hz, H-22), 7.05 (1H, d, *J* = 1.1 Hz, H-18), 7.12 (1H, d, *J* = 8.7 Hz, H-16), 7.75 (1H, d, *J* = 2.0 Hz, H-13), 7.77 (1H, dd, *J* = 8.7, 2.0 Hz, H-15), 12.41 (s, 5-OH); ^13^C NMR (125 MHz, DMSO-*d*_6_) δ 56.1 (OCH_3_), 60.5 (C-23), 76.3 (C-11), 78.9 (C-10), 94.0 (C-8), 98.7 (C-6), 103.5 (C-4a), 112.2 (C-18), 115.7 (C-21), 116.6 (C-13), 117.2 (C-16), 121.0 (C-22), 121.6 (C-15), 124.1 (C-14), 127.6 (C-17), 136.7 (C-3), 143.8 (C-12a), 145.4 (C-16a), 146.1 (C-2), 147.5 (C-20), 148.1 (C-19), 156.6 (C-9), 161.1 (C-5), 164.5 (C-7), 176.4 (C-4); EIMS *m*/*z* 480 [M]^+^; HREIMS *m*/*z* 480.1057, (calcd for C_25_H_20_O_10_, 480.1056).

### 3.5. Tyrosinase Inhibitory Activity

Mushroom tyrosinase (Sigma-Aldrich Co., St. Louis, MO, USA) was used for in vitro assays with slight modifications from a previously described method [42]. In this experiment, potassium phosphate buffer (0.07 mL, 50 mM) at pH 6.5, 0.03 mL tyrosinase (333 units/ mL), and 2 μL of the non-irradiated and irradiated samples (0–200 μg/mL) and the tested compounds (0–500 μM), were dissolved in absolute ethanol and inserted into 96-well plates. After 5 min incubation at room temperature, 0.1 mL _L_-tyrosine (2 mM) was added and incubated for additional 20 min. The optical density of the samples was measured at 475 nm (SpectraMax M2 Multi-Mode Microplate Reader, Molecular Devices, Sunnyvale, CA, USA) and showed a linear color change with time during the 20 min of the experiment compared to the control containing methanol (2 μL) and without inhibitor. Arbutin (Sigma-Aldrich Co., St. Louis, MO, USA), the known tyrosinase inhibitor, was used in the assays for comparison.

### 3.6. Molecular Docking Analyses

The X-ray crystal structure of mushroom tyrosinase (Agaricus bisporus) complexed with tropolone (PDB ID: 2Y9X) was obtained from the RCSB PDB (http://www.rcsb.org/pdb, accessed on 1 October 2024) was prepared using the protein preparation of the Discovery Studio software ver. 21.0.1.20298 (BIOVIA/Accelrys Inc., San Diego, CA, USA). The A chain of this protein was prepared for docking, the water molecules and tropolone were removed, and the hydrogens were added. The 2D structures of ligands were obtained from CAS SciFinder (American Chemical Society, Columbus, OH, USA) and then prepared in their energy minimized structures using the MM2 calculation of ChemOffice software (Chem3D Ultra 7.0, CambridgeSoft, Cambridge, MA, USA). The 3D structures for docking were prepared using the default values that exclude the production of isomer and tautomer in the ligand preparation tool of the Discovery Studio software. Protein–ligand simulations were performed using CDOCKER docking protocol in Discovery Studio software (BIOVIA/Accelrys Inc., San Diego, CA, USA), which is a molecular dynamics code based on the Chemistry at Harvard Macromolecular Mechanics (CHARMm) algorithm [43]. The grid box was generated by controlling the grid size such that it was sufficient to accommodate the selected compounds from the prepositioned tropolone as centroid. The active sites were constituted by residues His61, Cys83, His85, His94, His259, His263, His296, Asn260, His263, Phe264, Met280, Val283, Ala286, Cu401, Asp336, Gln351, Asp353, and Asp312. The best poses of each compound were selected based on –CDOCKER energy, a score including internal ligand strain energy and receptor–ligand interaction energy, as well as the interactions of critical residues within the docked position.

### 3.7. Statistical Analysis

The samples were evaluated in triplicate. The results were subject to variance analysis using SigmaPlot^TM^ 10.0.1 (Systat Software GmbH, Frankfurt am Main, Germany). Differences were considered significant at *p* < 0.05.

## 4. Conclusions

We established a novel method for the formation of isosilandrin (**2**) and 2,3-dehydrosilybin (**3**) through γ-irradiation on silybin. The optimal γ-ray dose for obtaining both radiolysis products in high yield is 300 kGy. In the evaluation of their tyrosinase inhibitory activity, compounds **2** and **3** showed approximately 1.8 and 4.5 times more inhibition than the non-irradiated silybin. A molecular docking analysis of the selected stereostructures of compounds **1**–**3** revealed stable binding energy levels. Among them, the stereostructures for compound **3** fit within the active sites of mushroom tyrosinase in the lowest energy conformations characterized by the phenylpropane and flavonol structures having optimal attractive electrostatic interactions with the catalytic triad residues of tyrosinase. Therefore, the bioactivity of silybin could be significantly enhanced through structural modifications using ionizing radiation technology, and compound **3** could be identified as a potent tyrosinase inhibitor given its ability to bind directly to the active sites of tyrosinase.

## Figures and Tables

**Table 1:** The relative contents of peak 1–3 (% of peak area at 240 nm) ^1^.

Peaks	Irradiated Dose			
	50 kGy	100 kGy	200 kGy	300 kGy
1	68.9 ± 5.8	56.2 ± 3.8	35.3 ± 4.3	16.7 ± 2.2
2	22.5 ± 2.1	28.4 ± 4.5	30.1 ± 2.7	33.5 ± 5.1
3	8.6 ± 1.8	15.5 ± 2.4	34.6 ± 1.5	49.8 ± 6.3

^1^ All samples were examined in a set of experiments repeated three times.

**Table 2:** Mushroom tyrosinase inhibitory activity of non-irradiated (0 kGy) and irradiated samples (50–300 kGy).

Dose of Irradiated Samples ^1^	Concentration (μg/mL)	Inhibition of Tyrosinase (%)	IC_50_ (μg/mL) ^2^
0	50	5.2 ± 0.2	>200
	100	11.4 ± 0.5	
	200	26.5 ± 0.8	
50	50	16.1 ± 0.4	>200
	100	23.6 ± 0.2	
	200	30.2 ± 1.0	
100	50	16.0 ± 0.5	>200
	100	20.9 ± 1.2	
	200	45.6 ± 1.8	
200	50	17.1 ± 0.7	198.1
	100	20.3 ± 0.2	
	200	52.2 ± 0.4	
300	50	17.3 ± 0.1	171.1
	100	30.8 ± 1.1	
	200	57.8 ± 1.5	
Arbutin ^3^	2300	50.0 ± 2.1	2300

^1^ Sample solution (1 g silybin in 100 mL MeOH in 10% DMSO) divided into 100 vials were irradiated with 0–300 kGy (absorbed dose). ^2^ All samples were examined in a set of experiments repeated three times. ^3^ Arbutin was used as a positive control.

**Table 3:** Mushroom tyrosinase inhibitory activities of compounds **1**–**3**.

Compounds	Tyrosinase (IC_50_, μM) ^1^
silybin (**1**)	>500
isosilandrin (**2**)	274.6 ± 12.5
2,3-dehydrosilybin (**3**)	109.5 ± 8.7

^1^ All compounds were tested in a set of experiments repeated three times.

**Table 4:** Docking score and interactions of the selected structures of **1**–**3** against tyrosinase.

Compound Name	Structure	-CDOCKER Energy (kcal/mol)	Interacting Residues
Arbutin ^1^	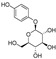	5.0142	His259, His263, Asn260, His244, Val248, Cu401
(+)-silybin A	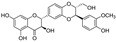	19.1086	Met280, Ala286, His263, Val283, Pro284, Phe264, Gly281, Ser282, Asn260, Gly281, Cu401
(−)-silybin B	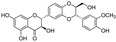	21.5132	Met280, Ala286, His263, Val283, Arg268, Phe264, Gly281, Asn260, Val248, His244, Cu401
(−)-isosilandrin A	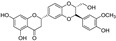	23.3343	Met280, Ala286, His263, Val283, Ser282, His85, Val248, Phe264, Leu275
(+)-isosilandrin B	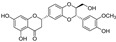	22.0735	Met280, Ala286, His263, Val283, Ser282, His85, Val248, Phe264
(−)-dehydrosilybin	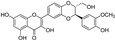	36.4082	Val283, His259, Met280, His263, Phe264, Arg268, Gly281
(+)-dehydrosilybin B	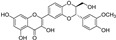	34.7141	Val283, His259, Met280, His263, Phe264, Arg268, His244, Val248

^1^ Arbutin was used as a positive control.

## Data Availability

The data presented in this study are available upon request from the corresponding author.
